# Simple and Robust Analysis of Cefuroxime in Human Plasma by LC-MS/MS: Application to a Bioequivalence Study

**DOI:** 10.1155/2014/981624

**Published:** 2014-04-24

**Authors:** Xingjiang Hu, Mingzhu Huang, Jian Liu, Junchun Chen, Jianzhong Shentu

**Affiliations:** Research Center for Clinical Pharmacy, State Key Laboratory for Diagnosis and Treatment of Infectious Diseases, First Affiliated Hospital, Zhejiang University, Qingchun Road 79, Hangzhou 310003, China

## Abstract

A simple, robust LC-MS/MS assay for quantifying cefuroxime in human plasma was developed. Cefuroxime and tazobactam, as internal standard (IS), were extracted from human plasma by methanol to precipitate protein. Separation was achieved on a Zorbax SB-Aq (4.6 × 250 mm, 5 **μ**m) column under isocratic conditions. The calibration curve was linear in the concentration range of 0.0525–21.0 **μ**g/mL (*r* = 0.9998). The accuracy was higher than 90.92%, while the intra- and interday precision were less than 6.26%. The extraction procedure provides recovery ranged from 89.44% to 92.32%, for both analyte and IS. Finally, the method was successfully applied to a bioequivalence study of a single 500 mg dose of cefuroxime axetil in 22 healthy Chinese male subjects under fasting condition. Bioequivalence was determined by calculating 90% Cls for the ratios of *C*_max_, AUC_0−*t*_, and AUC_0−*∞*_ values for the test and reference products, using logarithmic transformed data. The 90% Cls for the ratios of *C*_max_ (91.4%~104.2%), AUC_0−*t*_ (97.4%~110.9%), and AUC_0−*∞*_ (97.6%~111.1%) values were within the predetermined range. It was concluded that the two formulations (test for capsule, reference for tablet) analyzed were bioequivalent in terms of rate and extent of absorption and the method met the principle of quick and easy clinical analysis.

## 1. Introduction

Cefuroxime is a second-generation cephalosporin used against a variety of infections. Due to its low oral bioavailability, cefuroxime is administered orally as a prodrug in the form of cefuroxime axetil [1]. Upon administration, the acid-stable lipophilic prodrug undergoes hydrolysis to yield cefuroxime [2]. However, the oral bioavailability of this ester prodrug would be changed violently for suffering from many factors, such as food [3]. To be able to optimize the dosing, it is necessary to characterize the pharmacokinetics of cefuroxime which requires a selective and sensitive analytical method for cefuroxime in plasma.

Several methods, including HPLC-DAD, LC-MS/MS, and UPLC-MS/MS, had been reported for the determination of cefuroxime in human plasma. However, they all need a complicated and expensive sample pretreatment method, or solid-phase extraction [4–6], or protein precipitation combined with back-extraction [7, 8], or protein precipitation followed by supernatant evaporated [9], for cleanup and enrichment of plasma samples, so as to get a lower limit of quantification. To the best of our knowledge, there was only one method with LLOQ of 25 ng/mL using simple protein precipitation extraction [10]. Generally speaking, using LC-MS technique for quantification in biofluids, IS should have similar physical, chemical, and chromatographic properties as the analyte (ideally eluted at similar retention time) [11]. Nevertheless, in this literature, the retention time of cefuroxime and IS was far apart, as 8 min and 4.4 min, respectively. Thus, it could not compensate for the sample losses that might occur during the sample preparation and chromatographic steps as well as for matrix effects under certain conditions.

In this study, we designed a sensitive and robust LC-MS/MS method following simple protein precipitation extraction with tazobactam as IS for determination of cefuroxime in human plasma. This method was accurate, sensitive, robust, and simple and was successfully applied to a bioequivalence study of a single 500 mg dose of cefuroxime axetil formulations (test and reference) in 22 healthy Chinese male subjects under fasting condition.

## 2. Experimental

### 2.1. Chemicals and Reagents

**Figure 1:**
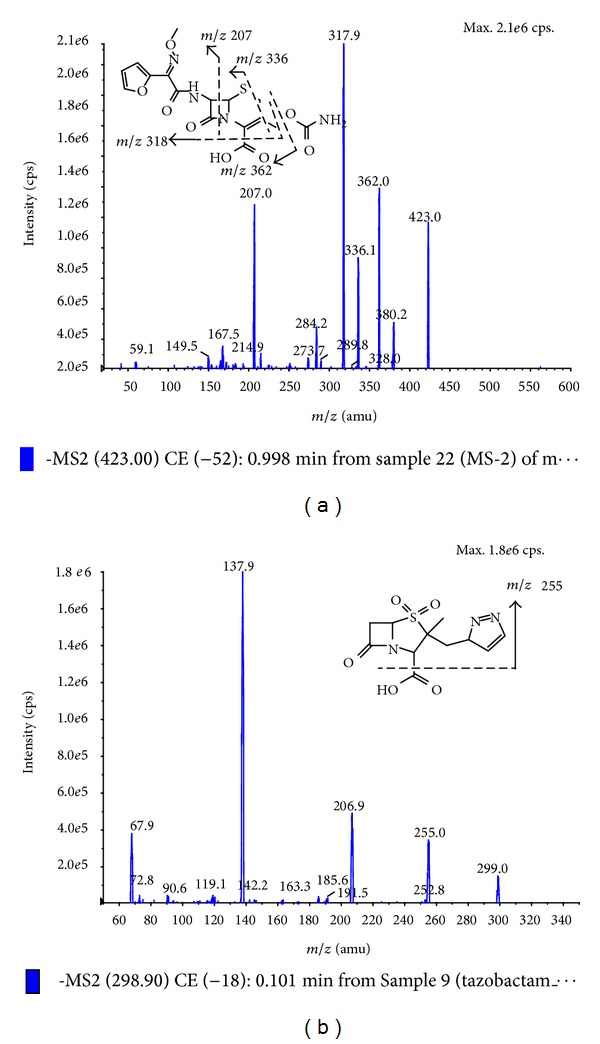
The structures and MS spectrums of cefuroxime (a) and IS (b).

Cefuroxime (*Batch No. 130493-200704, purity 91.4%*) and tazobactam (IS) (*Batch No. 130511-200402, purity 99.1%*) were supplied by the National Pharmaceutical Institute of China. The chemical structures are shown in Figure 1. HPLC grade methanol, acetonitrile were purchased from Merck KGaA Company (Darmstadt, Germany). Water was purified using a Milli-Q system (Milford, MA, USA). HPLC grade ammonium formate and formic acid were purchased from Sigma (St. Louis, MO, USA). Human plasma was obtained from the Blood Center of Zhejiang Province (Hangzhou, China).

### 2.2. Instruments

The HPLC was performed on an Agilent 1200 system equipped with a G1367C autosampler, a G1379B degasser, a G1316B thermostatted column, and a G1312B binary pump (Agilent, Waldbronn, Germany). The HPLC system was coupled with an API 4000 triple-quadrupole mass spectrometer (Applied Biosystems, Concord, ON, Canada) via electrospray ionization interface for mass analysis and detection. Data acquisition was performed with Analyst 1.4.2 software (Applied Biosystems).

### 2.3. LC-MS/MS Conditions

Separation was performed on an Agilent Zorbax SB-Aq (4.6 × 250 mm, 5 *μ*m) at a column temperature of 30°C. An isocratic mobile phase consisting of methanol/0.05% formic acid in water (42 : 58, v/v) was used at a flow rate of 1 mL/min, with the injection volume of 2 *μ*L. The autosampler was set at 4°C. A primary flow rate of 1 mL/min was split to 500 *μ*L/min using a T-piece. All measurements were carried out with mass spectrometer operated under the negative ESI mode. The multiple reaction monitoring transitions were* m/z *423.0 → 317.9 for cefuroxime and* m/z *298.9 → 138.0 for IS. Other parameters were as follows: collision gas, curtain gas, ion source gas 1 and ion source gas 2 (nitrogen) 6, 15, 55, and 50 psi, respectively; dwell time 200 ms; ion spray voltage −4500 V; ion source temperature 400°C; declustering potential (DP) −40 V for cefuroxime and −34 V for IS; collision energy −10 V for cefuroxime and −22 V for IS; collision exit potential (CXP) −10 V for cefuroxime and −9 V IS; and entrance potential (EP) −10 V for cefuroxime and IS. Unit resolution was used for both Q1 and Q3 mass detection.

### 2.4. Preparation of Standard Solution and Quality Control (QC) Samples

Stock solution (1.05 mg/mL) of cefuroxime was prepared in 50% methanol and was further diluted with 50% methanol to achieve standard working solutions at concentrations of 210, 105, 52.5, 21.0, 10.5, 5.25, 2.10, 1.05, and 0.525 *μ*g/mL. The QC stock solutions (low: 0.840 *μ*g/mL, medium: 16.8 *μ*g/mL, and high: 168 *μ*g/mL) were also prepared in the same way. Tazobactam (IS), stock solution 1.86 mg/mL prepared in 50% methanol, was diluted with 50% methanol to give a final concentration of 18.6 *μ*g/mL. Both of these stock solutions were stored at 4°C avoiding light for using.

The standard working solutions (20 *μ*L) were used to spike blank plasma samples (200 *μ*L). The final concentrations of cefuroxime standard calibration plasma samples were 21.0, 10.5, 5.25, 2.10, 1.05, 0.525, 0.210, 0.105, and 0.0525 *μ*g/mL, respectively. The QC samples were also prepared in the same way by adding 20 *μ*L diluted QC stock solutions to 200 *μ*L blank human plasma. The final concentrations of cefuroxime in the low-, medium-, and high-QC plasma samples were 0.0842 *μ*g/mL, 1.68 *μ*g/mL, and 16.8 *μ*g/mL, respectively.

### 2.5. Sample Extraction Procedures

After frozen human plasma samples were thawed at ambient temperature and adequately vortexed, a total of 200 *μ*L aliquot plasma sample was added with 20 *μ*L of 50% methanol (supplementary volume) and 20 *μ*L IS (18.6 *μ*g/mL) solution. After a thorough vortex mixing for 30 s, mixtures were precipitated with 600 *μ*L methanol, vortex-mixed for 30 s, and centrifuged at 13000 rpm for 5 min. Finally, 2 *μ*L of supernatant was injected into the LC-MS/MS system.

### 2.6. Method Validation

The current method was validated prior to the analysis of human plasma samples according to the guidance of bioanalytical method validation [12]. The selectivity, linearity, precision, accuracy, sensitivity, recovery, matrix effect, and stability of cefuroxime in plasma sample were assessed and investigated.

To evaluate selectivity, drug-free plasma samples from 6 individuals were analyzed to check for the presence of any interfering peaks at the elution times of both cefuroxime and IS. The calibration curves were constructed using 9 standards ranging in concentration from 0.0525 to 21.0 *μ*g/mL. The validity of the linear regression equation was indicated by the correlation coefficient (*r*).

The intraday and interday precision and accuracy were evaluated by assessing QC samples at the following concentrations (*n* = 6): LLOQ (0.0525 *μ*g/mL), low (0.0842 *μ*g/mL), medium (1.68 *μ*g/mL), and high (16.8 *μ*g/mL).

The extraction recovery and matrix effect of cefuroxime for three concentrations of QC samples was determined by comparing the response of analyte spiked plasma after extraction to that of analyte spiked into the solution extracted from blank plasma and the response of analyte spiked after extraction to that of analyte dissolved in mobile phase, respectively.

Stability experiments involved leaving the untreated plasma sample at ambient temperature for 6 h without light, placing the treated plasma sample in an autosampler for 20 h, three freeze-thaw cycles from −20°C to 25°C, and storing for 145 days at −20°C, using three aliquots of each QC sample at three different concentrations.

### 2.7. Application of the Assay

The method described in this paper was applied to a bioequivalence study of two oral formulations of cefuroxime axetil (test formulation, a 250 mg cefuroxime axetil capsule from a Chinese company; reference formulation, a 250 mg cefuroxime axetil tablet produced by GlaxoSmithKline, UK). The study followed a single dose, two-way randomized crossover design with a 1-week washout period between doses. After an overnight fast of at least 10 h, subjects received a single oral 500 mg dose of either the test or reference formulation with 240 mL of water. During both treatment periods, heparinized blood samples were collected at the following times: before (0.0 h) and at 0.33, 0.67, 1.0, 1.5, 2.0, 2.5, 3.0, 4.0, 5.0, 6.0, 8.0, and 10.0 h after dosing. The blood samples were centrifuged at 4000 rpm for 10 min, and plasma samples were separated and stored at −20°C until analyzed.

In addition to *C*_max⁡_ and *T*_max⁡_ obtained directly from the measured data, other PK parameters (AUC_0–*t*_, AUC_0–*∞*_, and *t*_1/2_) were calculated by noncompartmental analysis using Drug Statistics (DAS) software 2.1.1 (University of Science and Technology, Hefei, China). The relative bioavailability (*F%*) of the tested formulation was calculated as follows: *F*% = AUC_0–*t*_(test)/AUC_0–*t*_(reference) × 100%. Analysis of variance (ANOVA) using DAS 2.1.1 was performed on *C*_max⁡_, AUC_0–*t*_, and AUC_0–*∞*_ values evaluating treatment, period, sequence, and subject within sequence effects. Their ratios (test* versus *reference) of log-transformed data were analyzed for relative bioavailability. The 90% Cls served as interval estimates and were determined by two 1-sided *t*-tests. If the differences in PK parameters between the two formulations were not statistically significant (*P* > 0.05) and the 90% Cls for the ratios of *C*_max⁡_, AUC_0–*t*_, and AUC_0–*∞*_ are located within the bioequivalence criteria range (80~125% for AUC and 70~143% for *C*_max⁡_), then the two formulations were considered to have met the regulatory requirement for bioequivalence.

## 3. Results and Discussion

### 3.1. Method Development

**Figure 2:**
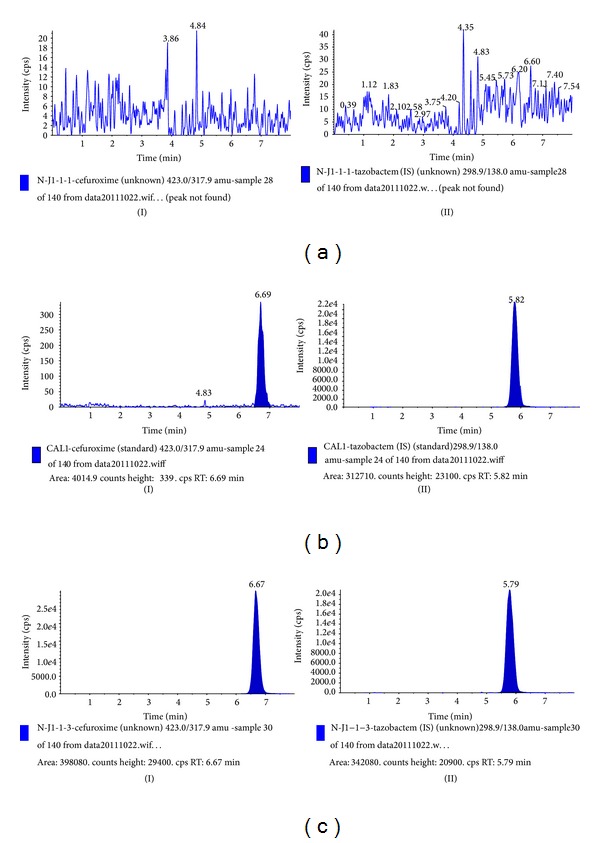
MRM chromatograms of cefuroxime (I) and IS (II) obtained from human plasma samples: (a) blank plasma, (b) blank plasma spiked with standard solution (LLOQ), and (c) plasma sample from a healthy subject 0.67 h after oral administration.

To optimize chromatography, stationary phase, the composition of mobile phase, and column temperature were investigated in the LC domain, so as to achieve optimal peak shape and good separation from the void volume. Because of their amphotericity of chemical properties, various available columns of different lengths and bonded phases (Zorbax SB-C_18_, Zorbax SB-Aq, Hypersil GOLD Aq, and Atlantis T3) were carefully evaluated. Finally, Agilent SB-Aq column was chosen in the present study for its high efficiency and peak symmetry. Different mobile phases (methanol-water and acetonitrile-water with different additives, such as formic acid and ammonium formate) were examined to obtain efficient chromatography and relatively short run time for cefuroxime and IS. It was found that the addition of formic acid could remarkably improve the peak symmetry and ionization of cefuroxime and IS. When methanol was used as the organic phase, the peak of cefuroxime was further improved. Therefore, the mobile phase was selected as methanol-mixture of 0.05% formic acid in water to achieve better separation and less interference from other components in the plasma. The retention time for cefuroxime and IS was 6.8 and 5.9 min, respectively. The total chromatographic run time was 8.0 min (Figure 2).

### 3.2. Selectivity

The typical MRM chromatograms of mixed blank plasma from six drug-free individuals, a spiked plasma sample with cefuroxime at LLOQ and IS, and a plasma sample from a healthy volunteer 0.67 h after an oral administration were shown in Figure 2. The results indicated that there was no apparent endogenous interference for the determination of cefuroxime.

### 3.3. Linearity of Calibration Curves and LLOQ

The standard calibration curve for spiked human plasma containing cefuroxime was linear over the range 0.0525–21.0 *μ*g/mL. Good linearity was observed for the analyte using a weighted (1/*x*) least squares linear regression analysis with a coefficient of determination *r* = 0.9998. Typical equations for the calibration curve were as follows: *Y* = (0.186 ± 0.002)*X* + (0.00024 ± 0.00049) (*n* = 3), where *X* represents the plasma concentration of cefuroxime (*μ*g/mL) and *Y* represents the ratios of cefuroxime peak area to that of IS. LLOQ under the optimized conditions was 0.0525 *μ*g/mL for cefuroxime, which was judged from the fact that the precision and accuracy were less than 20% (Table 1) and the *S*/*N* ratios were much higher than 10. The LLOQ was sufficient for the bioequivalence study of cefuroxime following an oral administration.

### 3.4. Precision and Accuracy

QC samples at three concentration levels were calculated over three validation runs (once a day). Six replicates of each QC level were determined in each run. Table 1 summarized the intraday and interday precision and accuracy for cefuroxime. In this assay, the intraday precision that was expressed by relative standard deviation (RSD) was no more than 2.84% for all tested concentrations (0.0842, 1.68, and 16.8 *μ*g/mL), and the interday precision was less than 6.26%. The accuracy ranged from 90.92% to 101.8%. The above values were within the acceptable range, which demonstrated the good stability and repeatability of this described method.

### 3.5. Recovery and Matrix Effect

The recoveries of the protein precipitation for cefuroxime were 89.44 ± 4.66%, 91.94 ± 0.94%, and 91.39 ± 1.67% at concentrations of 0.0842, 1.68, and 16.8 *μ*g/mL, respectively. Mean recovery for the IS was 92.32 ± 0.90%. The RSDs for all recoveries were less than 5.21% throughout the entire concentration ranges, indicating assay consistency.

The matrix effect was evaluated to determine the influence of matrix components on analyte quantification. Average matrix effect values obtained were 110.6 ± 5.10%, 109.8 ± 1.58%, 111.4 ± 2.12%, and 108.8 ± 1.52% for QC samples at concentrations of 0.0842, 1.68, and 16.8 *μ*g/mL, and IS. The results obtained were well within the acceptable limit [12] and indicated that the analysis of cefuroxime was not interfered with by endogenous substances in plasma.

### 3.6. Stability

The stability experiment was performed by using QC samples at concentrations of 0.0842, 1.68 and 16.8 *μ*g/mL, except for long-term stability for 0.105, 1.68 and 16.8 *μ*g/mL. The results indicated that cefuroxime was stable in untreated plasma when placed in the short-term (6 h) at room temperature, repeated three freeze/thaw cycles and stored at −20°C for 145 days. In addition, it was found also stable in treated-plasma samples when placed in autosampler at 4°C for 20 h (Table 2).

### 3.7. Bioequivalence Evaluation

**Figure 3:**
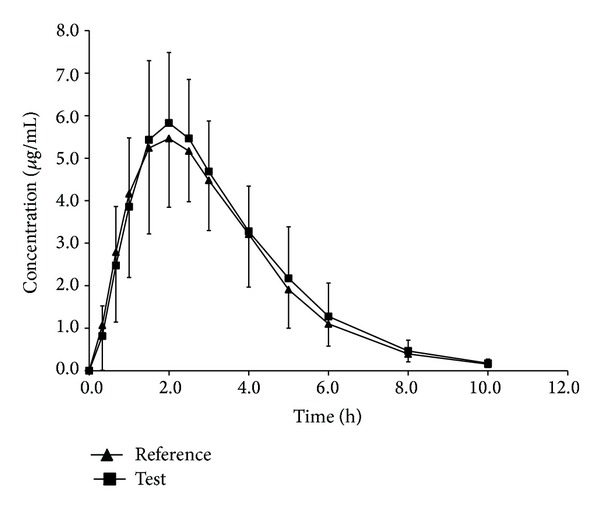
Mean plasma concentration-time profile of cefuroxime after oral administration of test and reference formulations to 22 healthy male subjects.

The mean plasma concentration-time curves of cefuroxime after oral administration of a single 500 mg dose of test and reference formulations in 22 healthy Chinese male volunteers were shown in Figure 3. The PK parameters of cefuroxime after oral administration of 500 mg test and reference formulations to 22 healthy volunteers were presented in Table 3. The results of the analysis of ANOVA for assessment of product, group, and period effects and 90% Cls for the ratio of *C*_max⁡_, AUC_0–*t*_, and AUC_0–*∞*_ values of test and reference products, using logarithmic transformed data, were shown in Table 3. Power of statistical test was 97.6% for *C*_max⁡_, 103.9% for AUC_0–*t*_, and 104.1% for AUC_0–*∞*_.

No significant differences in AUC_0–*t*_ or *C*_max⁡_ were found between the test and reference formulations. The multivariate analysis accomplished through analysis of variance revealed the absence of period, group, and product effects for AUC_0–*t*_, AUC_0–*∞*_, or *C*_max⁡_. The 90% Cls for the ratio of *C*_max⁡_ (91.4%~104.2%), AUC_0–*t*_ (97.4%~110.9%), and AUC_0–*∞*_(97.6%~111.1%) values for the test and reference products were all located within the bioequivalence criteria range (80~125% for AUC and 70~143% for *C*_max⁡_), proposed by China Food and Drug Administration [13]. It was concluded that the two formulations analyzed were bioequivalent in terms of rate and extent of absorption and, thus, may be used interchangeably, with no effect on therapeutic effect.

## 4. Conclusion

A simple and sensitive LC-MS/MS method for the quantification of cefuroxime in human plasma was developed. Method validation has been demonstrated by a variety of tests for selectivity, linearity, sensitivity, precision, recovery, matrix effect, and stability. This method is attractive for the pharmacokinetic and bioequivalence analysis of cefuroxime, because of its high sensitivity and accuracy.

## Figures and Tables

**Table 1:** Intraday and interday precision and accuracy of cefuroxime in human plasma.

	QC levels	Concentration (*μ*g/mL)	Mean concentration found (*μ*g/mL)	Precision (RSD%)	Accuracy (%)
Intraday (*n* = 6)	LLOQ	0.0525	0.0503	2.72	95.58
	L	0.0842	0.0766	2.84	90.92
	M	1.68	1.68	1.47	99.64
	H	16.8	16.7	1.40	99.43

Interday (3 days, *n* = 6)	L	0.0842	0.0851	6.26	101.1
	M	1.68	1.71	2.19	101.8
	H	16.8	16.8	2.09	100.1

**Table 2:** Summary of the stability of cefuroxime in human plasma on different conditions (*n* = 3).

Stability conditions	Concentration (*μ*g/mL)	Calculated concentration		
		Mean ± SD (*μ*g/mL)	Accuracy%	RSD%
Short-term (6 h, 25°C)	0.0842	0.0799 ± 0.0022	94.97	2.75
	1.68	1.70 ± 0.025	100.8	1.47
	16.8	16.4 ± 0.06	97.63	0.40

Long-term (145 days, −20°C)	0.105	0.103 ± 0.004	98.26	4.22
	1.68	1.73 ± 0.038	103.0	2.25
	16.8	16.77 ± 0.40	99.94	2.36

Autosampler (20 h, 4°C)	0.0842	0.0823 ± 0.0030	97.76	3.89
	1.68	1.71 ± 0.025	101.8	1.47
	16.8	16.6 ± 0.27	98.79	1.63

Three freeze-thaw cycles (from 25°C to −20°C)	0.0842	0.0837 ± 0.0048	99.46	5.76
	1.68	1.72 ± 0.032	102.1	1.84
	16.8	16.6 ± 0.36	98.65	2.15

**Table 3:** Mean pharmacokinetic parameters for cefuroxime after oral administration of 500 mg of test and reference formulations to healthy human volunteers under fasting condition (*n* = 22).

Parameters (units)	Reference formulation Mean ± SD	Test formulation Mean ± SD	Point estimate (90% Cls)
*T*_max⁡_ (h)	2.14 ± 0.85	2.25 ± 0.95	—
*C*_max⁡_ (*μ*g/mL)	6.42 ± 1.19	6.31 ± 1.45	97.6 (91.42–104.2)
*T*_1/2_ (h)	1.33 ± 0.10	1.38 ± 0.16	—
AUC_0–*t*_ (*μ*g·h/mL)	22.01 ± 3.95	23.02 ± 4.78	103.9 (97.4–110.9)
AUC_0–*∞*_ (*μ*g·h/mL)	22.30 ± 4.00	23.36 ± 4.87	104.1 (97.6–111.1)
